# A two-phase study evaluating the relationship between Thimerosal-containing vaccine administration and the risk for an autism spectrum disorder diagnosis in the United States

**DOI:** 10.1186/2047-9158-2-25

**Published:** 2013-12-19

**Authors:** David A Geier, Brian S Hooker, Janet K Kern, Paul G King, Lisa K Sykes, Mark R Geier

**Affiliations:** 1 The Institute of Chronic Illnesses Inc, 14 Redgate Ct, Silver Spring, MD, USA; 2 Simpson University, Redding, CA, USA; 3 University of Texas Southwestern Medical Center at Dallas, Dallas, TX, USA; 4 CoMeD Inc, Silver Spring, MD, USA

**Keywords:** Autism, Ethylmercury, Merthiolate, Thimerosal, Thiomersal, Vaccine

## Abstract

**Background:**

Autism spectrum disorder (ASD) is defined by standardized criteria of qualitative impairments in social interaction, qualitative impairments in communication, and restricted and stereotyped patterns of behavior, interests, and activities. A significant number of children diagnosed with ASD suffer a loss of previously-acquired skills, which is suggestive of neurodegeneration or a type of progressive encephalopathy with an etiological pathogenic basis occurring after birth. To date, the etiology of ASD remains under debate, however, many studies suggest toxicity, especially from mercury (Hg), in individuals diagnosed with an ASD. The present study evaluated concerns about the toxic effects of organic-Hg exposure from Thimerosal (49.55% Hg by weight) in childhood vaccines by conducting a two-phased (hypothesis generating/hypothesis testing) study with documented exposure to varying levels of Thimerosal from vaccinations.

**Methods:**

A hypothesis generating cohort study was undertaken to evaluate the relationship between exposure to organic-Hg from a Thimerosal-containing Diphtheria-Tetanus-acellular-Pertussis (DTaP) vaccine in comparison to a Thimerosal-free DTaP vaccine administered, from 1998 through 2000, for the risk of ASD as reported in the Vaccine Adverse Event Reporting System (VAERS) database (phase I). A hypothesis testing case–control study was undertaken to evaluate the relationship between organic-Hg exposure from Thimerosal-containing hepatitis B vaccines administered at specific intervals in the first six months of life among cases diagnosed with an ASD and controls born between 1991 through 1999 in the Vaccine Safety Datalink (VSD) database (phase II).

**Results:**

In phase I, it was observed that there was a significantly increased risk ratio for the incidence of ASD reported following the Thimerosal-containing DTaP vaccine in comparison to the Thimerosal-free DTaP vaccine. In phase II, it was observed that cases diagnosed with an ASD were significantly more likely than controls to receive increased organic-Hg from Thimerosal-containing hepatitis B vaccine administered within the first, second, and sixth month of life.

**Conclusions:**

Routine childhood vaccination is an important public health tool to reduce the morbidity and mortality associated with infectious diseases, but the present study provides new epidemiological evidence supporting an association between increasing organic-Hg exposure from Thimerosal-containing childhood vaccines and the subsequent risk of an ASD diagnosis.

## Background

Over the past two decades, the prevalence of individuals diagnosed with autism spectrum disorder (ASD) has risen dramatically [1], and currently at least 1 in 88 children are diagnosed with ASD in the US [2]. ASD is defined by standardized criteria of qualitative impairments in social interaction, qualitative impairments in communication, and restricted and stereotyped patterns of behavior, interests, and activities [3]. Although these core features define an ASD, recent investigations have described many health, physical, or behavioral co-morbid conditions consistently associated with an ASD such as gastrointestinal disturbances, incontinence, sleep problems, eating disorders, behavioral problems, and sensory processing issues [4].

In further considering the initial clinical presentation of ASD, a significant number of children diagnosed with ASD suffer a loss of previously-acquired skills between 6 and 18 months of age with an estimated incidence of regression among individuals diagnosed with ASDs ranging from 15% to more than 62% of all cases [5]. The importance of postnatal loss of neurological function in those diagnosed with an ASD between 6 and 18 months of age, as observed in affected children who have regressed, is this phenomena is suggestive of neurodegeneration or a type of progressive encephalopathy with an etiological pathogenic basis occurring after birth [5]. To date, the etiology of ASD remains under debate. However, many studies suggest toxicity in individuals diagnosed with an ASD [6]. More specifically, recent evidence suggests that mercury (Hg) may be either a causal factor in or contributory to the brain pathology in ASD, possibly working synergistically with other toxic compounds or pathogens to produce the abnormal brain pathology observed in those diagnosed with an ASD [7].

Hg is a heavy metal that is widespread and persistent in the environment, and infants in the US are exposed to significant levels of environmental Hg through the air, water, and breast milk [8]. In addition to environmental Hg exposure and maternal exposures from the mother’s Hg body burden, dietary intakes, and Hg-containing pharmaceuticals administered to the mother while the child is developing in utero, and injected organic-Hg from Thimerosal-containing childhood vaccines that have been and, in many countries, remain a significant source of Hg exposure for many infants during the first six months of life.

Thimerosal is an organic-Hg compound (49.55% Hg weight) added to vaccines as a preservative, typically at concentrations from 0.005% to 0.01% (12.5 μg Hg or 25 μg Hg per 0.5 mL vaccine dose) [9]. Thimerosal is known to rapidly dissociate into ethyl-Hg chloride, ethyl-Hg hydroxide, and sodium thiosalicylate in saline solutions. As a result of adherence to the recommended routine childhood vaccination schedule in the US during the 1990s infants may have been exposed to bolus doses of Hg nominally ranging from 12.5 μg Hg to 62.5 μg Hg that collectively added up to nominally 200 μg Hg from Thimerosal-containing childhood vaccines during the first six months of life (3 Diphtheria-Tetanus-Pertussis vaccines with 25 μg Hg per dose + 3 Haemophilus influenzae type B vaccines with 25 μg Hg per dose + 3 hepatitis B vaccines with 12.5 μg Hg per dose + 1 influenza vaccine with 12.5 μg Hg per dose, > 50% of all Hg exposure when considering environmental sources of Hg). This dosing pattern continues unabated in many developing nations to the present day. Moreover, despite a call for the removal of Thimerosal from all vaccines in the United States on July 7, 1999 by the American Academy of Pediatrics and United States Public Health Service [10], many children in the US continue to receive significant doses of Hg from the routinely recommended administration of Thimerosal-containing influenza vaccines (where more than 50% of all doses of influenza vaccine continue to contain 0.01% Thimerosal) to pregnant women, infants, and young children [9].

The purpose of the present study was to evaluate concerns about the toxic effects of organic-Hg exposure from Thimerosal in childhood vaccines by conducting a two-phased study with documented exposure to varying levels of Thimerosal from vaccinations. In the first phase, a hypothesis generating study was undertaken to evaluate the relationship between exposure to organic-Hg from a Thimerosal-containing Diphtheria-Tetanus-acellular-Pertussis (DTaP) vaccine in comparison to a Thimerosal-free DTaP vaccine for the risk of ASD in the Vaccine Adverse Event Reporting System (VAERS) database (phase I). In the second phase, a hypothesis testing study was undertaken to evaluate the relationship between organic-Hg exposure from Thimerosal-containing hepatitis B vaccines administered at specific intervals and the risk of ASD in the Vaccine Safety Datalink (VSD) database (phase II).

## Methods

### Phase I (hypothesis generating) - VAERS database

The VAERS is an epidemiological database that has been maintained jointly by the US Centers for Disease Control and Prevention (CDC) and US Food and Drug Administration (FDA) since 1990 as a surveillance tool to evaluate vaccine safety. Specific adverse events following vaccination are required to be reported to this database as mandated by law, but other adverse events that occur following vaccine administration are passively reported to VAERS. The VAERS Working Group of the CDC has previously acknowledged that less than 5% of the total adverse events reported to VAERS are reported by parents. Specific serious adverse events and deaths reported to VAERS are followed-up by the CDC/FDA. The VAERS Working Group of the CDC and the FDA have repeatedly analyzed and published epidemiologic studies based upon VAERS [11,12].

The VAERS Working Group notes that VAERS is simple to use, flexible by design, and the data are available in a timely fashion, but it also warns that the potential limitations may include systematic error due to underreporting, erroneous reporting, frequent multiple exposures, multiple outcomes and lack of precise denominators. In addition, when evaluating data from VAERS, it is important to note that, for any reported event, no cause and effect relationship has been established. VAERS is interested in all potential associations between vaccines and adverse events. Therefore, VAERS collects information on any adverse event following vaccination, be it coincidental or truly caused by a vaccine [11,12].

An analysis of the VAERS updated through June 12, 2013 was undertaken using the CDC Wonder online computer interface (http://wonder.cdc.gov/vaers.html). This portal provides a direct method for independent investigators to rapidly analyze up-to-date data in VAERS.

### Determining the population at risk

The population at risk included infants receiving either Thimerosal-containing DTaP vaccines manufactured by Pfizer\Wyeth-Lederle Laboratories (ACEL-IMMUNE™) or Thimerosal-free DTaP vaccines manufactured by GlaxoSmithKline (INFANRIX™), both of which were administered in the United States from 1998 through 2000.

### Determining outcomes

The VAERS was searched for ASD adverse event reports with outcome symptoms of autism (VAERS code: 10003805) and autism spectrum disorder (VAERS code: 10063844) among those with a specified state of residency in the United States. Among the adverse event reports identified, only those associated with Thimerosal-containing DTaP vaccines manufactured by Pfizer\Wyeth-Lederle Laboratories (VAERS code: 234) or Thimerosal-free DTaP vaccine doses manufactured by GlaxoSmithKline (VAERS code: 286) administered in the United States from 1998 through 2000 were examined in the present study. Overall, it was determined that there were a total of 38 ASD adverse events reported to VAERS following Thimerosal-containing DTaP vaccine doses manufactured by Pfizer\Wyeth-Lederle Laboratories and a total of 17 ASD adverse events reported to VAERS following Thimerosal-free DTaP vaccine doses manufactured by GlaxoSmithKline. These numbers were used as the numerators in phase I.

### Determining exposure

The Biologics Surveillance Summaries (BSS) of the CDC, which are broken down by vaccine manufacturer, were used to determine the net number of doses of vaccine distributed (this number subtracts the number of doses returned to the manufacturer from the total number distributed) (personal communication from Lisa Galloway of the CDC on May 23, 2002). The applicable BSS reported a total of 14,794,210 net number of doses of the Thimerosal-free DTaP vaccine were distributed from 1998 through 2000 and a total of 16,335,650 net number of doses of the Thimerosal-containing DTaP vaccine were distributed from 1998 through 2000. These numbers were used as the denominators in phase I.

### Statistical analyses

The Fisher’s exact test contained in the StatsDirect (version 2.7.8) statistical software package was utilized for statistical analyses, and a two-sided p-value < 0.05 was considered to be statistically significant. A comparison of the frequency of ASD adverse events reported to VAERS per the total net number of doses for each vaccine type was completed. The null hypothesis was that, regardless of Thimerosal content of the vaccines under study, a similar frequency would be observed for ASD adverse events reported to VAERS per the total net number of doses for each vaccine type.

### Phase II (hypothesis testing) VSD database

The study protocol employed was approved by the CDC, the Institutional Review Board (IRB) of Kaiser Permanente North-West (KPNW), and the IRB of Kaiser Permanente Northern California (KPNC). The data were analyzed at the secure Research Data Center of the National Center for Health Statistics in Hyattsville, MD. The views expressed in this study do not necessarily reflect those of the CDC or those of Kaiser Permanente.

### Determining the population at risk

A cohort of infants enrolled in the VSD project (updated through the end of 2000) from KPNW, Kaiser Permanente Colorado (KPC), and KPNC were examined using SAS® software. The VSD project was created in 1991 by the National Immunization Program (NIP) of the CDC and VSD methods were previously described [13-15]. The project links medical event information, specific vaccine history, and selected demographic information from the computerized databases of several HMOs. The cohort examined was comprised of individuals with non-missing date of birth, non-missing gender, and were HMO enrolled from their date of birth.

### Determining cases

The outcome files (inpatient and outpatient diagnoses) from this population were then reviewed to find the first instance of International Classification of Disease, 9^th^ revision (ICD-9) diagnosed ASD (ICD-9 code: 299.00). If there were multiple instances of the same diagnosis in a child, only the first instance was counted. In addition, to ensure the potential for an association between exposure and outcome, only individuals diagnosed with an ASD following administration of all the vaccines under study were included in the present analyses as cases.

A total of 307 cases diagnosed with ASD (males = 249, females = 58, male/female ratio = 4.3:1) born between 1991 through 1999 were identified. These individuals diagnosed with ASD were evaluated to determine their mean age of initial diagnosis of ASD and the standard deviation of mean age of initial diagnosis of ASD (4.2 ± 1.54 years-old).

### Determining controls

In order to identify controls without an ASD diagnosis who would have only a minimal chance of receiving such a diagnosis, controls had to have been continuously enrolled from birth for at least 7.29 years (mean age of initial diagnosis of ASD + 2× standard deviation of mean age of initial diagnosis of ASD). Applying this follow-up criterion yielded a total of 25,632 controls without an ASD diagnosis (males = 13,110, females = 12,522, male/female ratio = 1.05) born between 1991 through 1993.

### Hepatitis B vaccine exposure

The vaccine file for cases and controls was then reviewed to determine the exact dates of hepatitis B vaccine administration. Those cases and controls receiving no doses of hepatitis B vaccine were also included in the present study. Overall among the cases and controls, Hg exposure was assigned as 12.5 μg organic-Hg per dose for those receiving a pediatric hepatitis B vaccine or 0 μg organic-Hg per dose for those receiving combined Haemophilus influenzae type b (Hib)-hepatitis B vaccine or for those receiving none of the aforementioned vaccines.

### Statistical analyses

The Fisher’s exact test contained in the SAS® software was utilized for statistical analyses, and a two-sided p-value < 0.05 was considered statistically significant. In the first case–control experimental group (Experiment I), the data was examined to determine the frequency of exposure to 12.5 μg organic-Hg from a Thimerosal-containing hepatitis B vaccine in the first month of life in comparison to the frequency to 0 μg organic-Hg from Thimerosal-containing hepatitis B vaccine in the first month of life among cases and controls. In the second case–control experimental group (Experiment II), the data was examined to determine the frequency receiving two Thimerosal-containing hepatitis B vaccines within the first two months of life or a total of 25 μg organic-Hg in comparison to the frequency receiving 0 μg organic-Hg from Thimerosal-containing hepatitis B vaccines in the first two months of life among cases and controls. In the third case–control experimental group (Experiment III), the data was examined to determine the frequency of receiving three Thimerosal-containing hepatitis B vaccines within the first six months of life or a total of 37.5 μg organic-Hg in comparison to the frequency of receiving 0 μg organic-Hg in the first sixth months of life from Thimerosal-containing hepatitis B vaccines among cases and controls. The overall null hypotheses for each of the case–control experimental groups examined was that there would be no difference in the frequency of exposure to organic-Hg doses from Thimerosal-containing hepatitis B vaccines among cases and controls.

## Results

### Phase I

Table 1 shows the relationship between ASD adverse events reported to VAERS following Thimerosal-containing DTaP vaccine administration in comparison to Thimerosal-free DTaP vaccine. It was observed that there was a 2.02-fold significantly (p < 0.02) increased risk ratio for the incidence of ASD reported following the Thimerosal-containing DTaP vaccine in comparison to the Thimerosal-free DTaP vaccine.

**Table 1:** **A summary of Thimerosal**-**containing DTaP vaccine administration in comparison to Thimerosal**-**free DTaP vaccine as a risk factor for ASD adverse event reports in the VAERS**

**Group examined**	**Number autism/Autism spectrum disorder events**	**Net number of doses distributed (1998–2000)**	**Risk ratio (95% CI)**	**p-value**
Thimerosal-Containing DTaP Vaccine	38	16,335,650		
			2.02 (1.15–3.56)	< 0.02
Thimerosal-Free DTaP Vaccine	17	14,794,210		

### Phase II

Table 2 displays the relationship between cases and controls being administered increasing doses of organic-Hg from Thimerosal-containing hepatitis B vaccines at several specific points within the first six months of life. It was observed that cases were significantly more likely (odds ratio = 2.18, p < 0.00001) than controls to receive 12.5 μg organic-Hg from Thimerosal-containing hepatitis B vaccine in comparison to 0 μg organic-Hg from Thimerosal-containing hepatitis B vaccine within the first month of life. It was also observed that cases were significantly more likely (odds ratio = 2.11, p < 0.0001) than controls to receive 25 μg organic-Hg from Thimerosal-containing hepatitis B vaccines in comparison to 0 μg organic-Hg from Thimerosal-containing hepatitis B vaccines within the first two months of life. Finally, cases were significantly more likely (odds ratio = 3.39, p < 0.001) than controls to receive 37.5 μg organic-Hg from Thimerosal-containing hepatitis B vaccines in comparison to 0 μg organic-Hg from Thimerosal-containing hepatitis B vaccines within the first six months of life.

**Table 2:** **A summary of organic**-**mercury exposure from Thimerosal**-**containing hepatitis B vaccine administration among ASD cases and controls in the VSD database**

**Group examined**	**Number of cases diagnosed with ASD (%)**	**Number of controls without an ASD (%)**	**Odds ratio (95% CI)**	**p**-**value**
***Experiment I***				
12.5 μg organic-mercury within 1st month	155 (50.49)	8,161 (31.84)		
			2.18 (1.74–2.73)	< 0.00001
0 μg organic mercury within 1st month	152 (49.51)	17,471 (68.16)		
***Experiment II***				
25 μg organic-mercury within first 2 months	154 (50.49)	8,172 (32.55)		
			2.11 (1.68–2.64)	< 0.0001
0 μg organic-mercury within first 2 months	151 (49.51)	16,935 (67.45)		
***Experiment III***				
37.5 μg organic-mercury within first 6 months	30 (76.92)	1,073 (49.54)		
			3.39 (1.60–7.18)	< 0.001
0 μg organic-mercury within first 6 months	9 (23.08)	1,093 (50.46)		

## Discussion

The present study evaluated the potential relationship between organic-Hg exposure from Thimerosal-containing childhood vaccines and ASD using a two-phase study design. In the first phase, the VAERS database was analyzed for hypothesis generation, and it revealed a significant association between increasing organic-Hg exposure from Thimerosal-containing DTaP vaccine administration in comparison to Thimerosal-free DTaP vaccine for the risk of ASD. In the second phase, the VSD database was analyzed for hypothesis testing, and it revealed that those cases diagnosed with an ASD were at significantly greater risk of exposure to increasing doses of organic-Hg from Thimerosal-containing hepatitis B vaccines administered at three specific intervals within the first six months of life in comparison to controls.

The overall two-phased epidemiological method to evaluate vaccine safety using the VAERS for hypothesis generation (phase I) and the VSD for hypothesis testing (phase II) was previously described by investigators from the CDC and the FDA [16-18]. Specifically, these investigators described that products, such as vaccines, which are intended for healthy people, must be held to a high standard of safety assurance. However, the study of vaccine risks is more complex than for therapeutic products because the exposure is virtually universal for many vaccines, ensuring the chance occurrence of many adverse outcomes in temporal association with vaccines. As a result, these investigators described a two-phased approach by first identifying vaccine safety risks through reports to VAERS (hypothesis generating), and then using the VSD, a consortium of managed care organization, to more rigorously evaluate vaccine-associated risks (hypothesis testing).

It is interesting to consider the specific methods employed to evaluate the potential relationship between organic-Hg exposure from Thimerosal-containing childhood vaccines and the risk of ASD in each of the two phases of the present study. Specifically, in phase I, differences in organic-Hg content were evaluated based upon the differences in Thimerosal content of two different DTaP vaccines manufactured by different companies (i.e., one used Thimerosal as a preservative and the other used 2-phenoxyethanol). Other than the difference in organic-Hg content based upon vaccine manufacturer, these vaccines were similar, being recommended for administration to US infants in the same vaccine schedule at 2, 4, and 6 months of age [19].

Specifically, in phase II, differences in cumulative doses of organic-Hg received at specific intervals during the infant period were evaluated based upon the wide-ranging recommendations for routine hepatitis B vaccine administration. In 1991, the Advisory Committee on Immunization Practices (ACIP) recommended that infants should receive their hepatitis B vaccine doses as follows: first dose between birth and 2 months of age, second dose between 1 and 4 months of age, and third dose between 6 and 18 months of age [20].

Importantly, all told, it is apparent that the differences in organic-Hg exposures observed in both phases of the present study were not the result of a small group of children receiving anomalous exposures to vaccines. Instead, in phase I, these differences were a function of vaccine composition by vaccine manufacturer. Alternatively, phase II assessed varying levels of organic-Hg exposure which resulted from the varying windows recommended for administration of hepatitis B vaccines during the first year of life.

The results observed in the present study also appear to offer important potential biological mechanistic insights into the relationship between the timing and cumulative effects of organic-Hg exposure from Thimerosal-containing vaccines and the risk of receiving a diagnosis of ASD. For example, the effects of later additional organic-Hg exposure from administration of a second or third Thimerosal-containing hepatitis B vaccine did not increase the risk of receiving a diagnosis of ASD in a dose-proportional manner (i.e., odds ratio for second dose of Thimerosal-containing hepatitis B vaccine = 4 and odds ratio for third dose of Thimerosal-containing hepatitis B vaccine = 6). Instead, the odds ratio of receiving an ASD diagnosis after receiving two doses of Thimerosal-containing hepatitis B vaccine (25 μg organic-Hg) by two months of age was similar to the of receiving one dose of Thimerosal-containing hepatitis (12.5 μg organic-Hg) by one month of age. The odds ratio for receiving an ASD diagnosis after three doses of Thimerosal-containing hepatitis B vaccine (37.5 μg organic-Hg) was increased relative to those children receiving one or two doses of Thimerosal-containing hepatitis B vaccine, but the increase observed was significantly below what which would be expected from a cumulative perspective without considering the variable of timing of administration. Our observation of odds ratio being modified by time of exposure is consistent with previous observations regarding other cases of Hg intoxication, in which early exposure is associated with more significant adverse effects (i.e., Hg intoxication susceptibility: fetuses > infants > children > adults) [21].

In addition, the results observed in the present study support the hypothesis that a significant number of children diagnosed with an ASD experience neurodegeneration or a type of progressive encephalopathy with an etiological pathogenic basis occurring after birth. This is the case because the source of exposure examined in the present study was from Thimerosal-containing childhood vaccines administered between birth and 6 months of age, and a significant number of children diagnosed with an ASD have been reported to suffer a loss of previously-acquired skills between 6 and 18 months of age [5]. Because those exposed to Thimerosal-containing vaccines have a statistically significant increased risk of being diagnosed with an ASD, and because administration of Thimerosal-containing vaccines examined in this study was administered between birth and 6 months of age, it is valid to suggest that a significant number of individuals examined in this study who were diagnosed with an ASD suffered neurodegeneration or a type of progressive encephalopathy for which the etiological pathogenic basis was exposure to organic-Hg from Thimerosal administered as a vaccine component.

Importantly, many recent studies support the biologically plausible role of organic-Hg exposure from Thimerosal-containing vaccines in the pathogenesis of ASD [9]. Investigators have examined the distribution of organic-Hg following administration of Thimerosal to animals and infants. For example, administering Thimerosal mimicking the US vaccine schedule of the 1990s to infant monkeys, researchers found that significant levels of Hg were present in the brain (about 40–50 parts-per-billion), and a significant fraction of the Hg was present as inorganic-Hg (about 16 parts-per-billion) that was observed to not significantly decline 120 days following the last dose of Thimerosal [22]. Other investigators undertook further evaluations of the speciation of Hg present in rat tissues following administration of Thimerosal [23]. Interestingly, these researchers observed that administration of Thimerosal resulted in significant brain levels of Hg with 63% present in the form of inorganic-Hg, 13.5% as ethyl-Hg, and, unexpectedly, 23.7% as methyl-Hg.

In addition, investigators observed that Thimerosal-containing vaccine administration to human infants significantly increased the vaccinated infants’ blood Hg levels (with some infants having total blood Hg levels in excess of the safety limits established by the US Environmental Protection Agency) and also significantly increased the vaccinated infants’ hair ethyl-Hg levels (with some infants having total hair Hg levels in excess of the safety limits established by the US Environmental Protection Agency of 1 part-per-million) [9]. Finally, in further research on the distribution of Hg species within the body, investigators recently demonstrated that ethyl-Hg is actively transported across neuronal cellular membranes to the same degree as methyl-Hg, by the L-type neutral amino acid carrier transport (LAT) system [24].

The presence of Hg within brain cells is significant because it can induce cellular damage consistent with the neuronal damage observed in the brains of individuals diagnosed with ASD, and this damage is dependent upon thiol availability, especially glutathione, in that inadequate thiol availability results in significantly greater cellular damage from Hg [7,25]. Many previous studies reveal children diagnosed with ASD have limited thiol availability and decreased glutathione reserve capacity, deficits which make them more susceptible to the toxic effects of Hg exposure from administration of Thimerosal-containing vaccines [9].

Studies have also evaluated the potential for organic-Hg exposure from Thimerosal-containing vaccine administration to induce ASD pathology or clinical symptoms in animal model systems. These studies have yielded significant pathology or clinical symptoms in mice, rats, hamsters, and monkeys that are consistent with those observed in ASD following exposure to Thimerosal-containing vaccines mimicking the 1990s US routine childhood vaccination schedule [9].

The results observed in the present study are also supported by a number of previous epidemiological studies finding an association between organic-Hg exposure from Thimerosal-containing vaccines and ASD, using several epidemiological methods in various databases. For example, Young et al., using an ecological study design, evaluated the relationship between the birth cohort prevalence of specific neurodevelopmental disorder and birth cohort exposures to Hg from Thimerosal-containing childhood vaccines in the VSD [26]. Consistent with the results observed in the present study, it was seen that infants receiving an additional 100 μg organic-Hg from Thimerosal-containing childhood vaccines from birth to 7 months of age, had a significantly increased risk ratio of 2.87 for ASD, and infants receiving an additional 100 μg organic-Hg from Thimerosal-containing childhood vaccines from birth to 13 months of age, had a significantly increased risk ratio of 2.62 for ASD.

As another example, Gallagher and Goodman evaluated the relationship between administration of Thimerosal-containing hepatitis B vaccine administration and the subsequent risk of individual being diagnosed with an ASD, based upon assessment of the National Health Interview Survey (NHIS) 1997–2002 data sets [27]. They reported that boys diagnosed with an ASD in comparison to controls had a 3-fold significantly greater odds ratio for receiving a Thimerosal-containing hepatitis B vaccine during the first month of life.

Previously, Geier and Geier reported on the results of a meta-analysis using statistical modeling to evaluate the relationship between exposure increased Hg doses from Thimerosal-containing vaccines and neurodevelopmental disorder adverse event reports in VAERS [28]. They reported that there was a 1.6-fold significantly increased risk of ASD adverse event reports submitted to VAERS following additional doses of organic-Hg from Thimerosal-containing vaccine administration.

The results of the present study differ from several other cohort studies that failed to find an association between ASD and organic-Hg exposure from Thimerosal-containing childhood vaccines [29-31]. This may have occurred, in part, because other studies examined cohorts with significantly different childhood vaccine schedules and with different diagnostic criteria for outcomes. This difference may have also occurred because these other studies employed different epidemiological methods, especially with respect to the issue of follow-up period for individuals in the cohorts examined. The method used to measure follow-up period for individuals is a critical issue in all studies examining the relationship between exposures and the subsequent risk of an ASD diagnosis. This is the case because the risk of an individual being diagnosed with an ASD is not uniform throughout his/her lifetime. As observed in the present study, the initial mean age for an ASD diagnosis was 4.2 years-old, and the standard deviation of mean age of the initial diagnosis of ASD was 1.54 years-old. Therefore, any follow-up method that fails to consider the lag-time between birth and age of initial ASD diagnosis will likely not be able to observe the true relationship between exposure and the subsequent risk of an ASD diagnosis.

This type of concern is apparent with respect to the study published by Hviid et al. [29]. These investigators reported on a population-based cohort study of all children born in Denmark from January 1, 1990, until December 31, 1996 comparing children vaccinated with a Thimerosal-containing vaccine formulation (early years of the study) with children vaccinated with a Thimerosal-free formulation (latter years of the study) of the same vaccine. In order to evaluate years of follow-up for individuals in the study, these investigators described them in terms of person-years. As a result, these investigators assumed equality of person-years of contribution to the study regardless the age of the individual, despite observing a similar mean age of an ASD diagnosis (4.7 ± 1.7 years-old) as the present study. The consequence in the Hviid et al. [29] study was that an out-of-proportion number of person-years of follow-up was observed for the older children who had received Thimerosal-containing vaccine formulation in comparison to the younger children vaccinated with a Thimerosal-free vaccine formulation, rendering the results of the study uninterpretable. Similar limitations apply to the cohort studies published by Andrew et al. [30] (median age of initial ASD diagnosis = 4.4 years-old) and Verstraeten et al. [31] (median age of initial ASD diagnosis HMO A = 49 months [4.08 years] and HMO B = 44 months [3.67 years]), which used hazard models (i.e., models which presume a constant relative hazard of ASD diagnosis over time) to evaluate follow-up of individuals in their respective examinations of the relationship between Thimerosal-containing vaccine exposures and the subsequent risk of an ASD diagnosis.

In order to further reveal the importance of follow-up period, the following example is informative. In the aforementioned Verstraeten et al. [31] study, the investigators examined a VSD dataset that shared significant overlap with the one examined in the present study. Verstraeten et al. [31] observed a risk ratio = 1.16 (95% confidence interval = 0.78-1.71) for ASD diagnosed among infants receiving 12.5 μg organic-Hg exposure from Thimerosal-containing vaccines administered in the first month of life. This observation is in contrast to the odds ratio = 2.18 (95% confidence interval = 1.74-2.73) observed in the present study when comparing cases to controls for receiving 12.5 μg organic-Hg exposure from Thimerosal-containing hepatitis B vaccine in comparison to receiving 0 organic-Hg exposure from Thimerosal-containing hepatitis vaccine administered in the first month of life. A further analysis of the dataset examined in the present study, using reduced lengths of continuous follow-up among controls presumed to be without an ASD diagnosis, revealed that if the length of continuous follow-up criteria was reduced to the mean age of initial ASD diagnosis + standard deviation of initial ASD diagnosis (5.7 years), the odds ratio = 1.69 (95% confidence interval = 1.35-2.12). Further, if the length of continuous follow-up criteria was reduced further among controls to only the mean age of initial ASD diagnosis (4.2 years), the odds ratio = 1.47 (95% confidence interval = 1.18-1.85). Finally, if the length of continuous follow-up criteria was reduced even further among controls to only the mean age of initial ASD diagnosis - standard deviation of initial ASD diagnosis (2.7 years), the odds ratio = 1.21 (95% confidence interval = 0.97-1.51), a value that, considering the confidence intervals, is consistent with the risk ratio = 1.16 (95% confidence interval = 0.78-1.71) observed in the Verstraeten et al. [31] study. Although the aforementioned odds ratios are still statistically significant (except for the last analysis requiring continuous follow-up criteria among controls to only the mean age of ASD diagnosis - standard deviation), the effect becomes less pronounced in each instance as increasing levels of ambiguity of diagnosis are introduced among controls due to the lack of appropriate follow-up.

### Strengths/limitations

A strength of the present study involved examination of two separate cohorts of children from two separate databases (i.e., VAERS and VSD). The observations made in VAERS were based upon retrospective assessment of passively reported adverse events. In contrast, the VSD observations were made based upon retrospective assessment of prospectively collected medical records of patients enrolled in various HMOs. Despite these intrinsic differences in data-collection methods between the two databases, in both databases organic-Hg exposure from Thimerosal-containing vaccine administration was consistently associated with a subsequent diagnosis of an ASD.

The study design used to evaluate the relationship between exposure and outcome was another significant strength of the present study. In phase I of the study, adverse event reports in VAERS were analyzed. The method employed to examine VAERS ensured that the exposures to the various types of vaccines studied occurred prior to the outcomes described in the adverse event reports, since those reporting the subsequent adverse outcomes in association with the vaccines listed on the adverse event reports. In phase II, cases had to be enrolled from birth and were required to be continuously enrolled until a medical diagnosis of an ASD was made and controls had to be enrolled from birth for a sufficient time period to ensure that there was a very small chance that, during additional follow-up, any of the controls would be medically diagnosed with an ASD. As a result, any factors associated with enrollment (i.e., adjustment for potential independent variables between cases and controls were not necessary because enrollment was from birth) or healthcare-seeking behavior (i.e., adjustment for potential access/availability of healthcare was continuous among cases and controls) were minimized.

For cases diagnosed with an ASD, it was possible to mathematically evaluate the mean and standard deviation of age for initial ASD diagnoses within the VSD. From this information, it was possible to estimate how many additional potential diagnoses of ASD were missed. It was decided, *a priori*, to ensure adequate amounts of data for our analyses, that controls had to be continuously enrolled in the VSD from birth until they were at least 7.29 years-old (mean age of initial diagnosis of ASD + 2× standard deviation of mean age of initial diagnosis of ASD). Based on the data for age of initial diagnosis for ASD, this was a sufficient period to ensure that, with further follow-up, those controls without an ASD would probably not receive an ASD diagnoses in the VSD (mathematically there is a < 2.5% chance of these individuals being diagnosed with an ASD with additional follow-up time beyond 7.29 years). In addition, cases diagnosed with an ASD were specifically evaluated to ensure that only those cases diagnosed with an ASD following vaccine administration were considered in the present analyses.

Another strength of the study was that the VSD and VAERS data were collected independently of the study design used in the present study. The VSD data records analyzed, in particular, were collected as part of the routine healthcare individuals received as part of the participation with their respective HMOs, and as such, the healthcare providers in no way were thinking about the potential association between vaccine exposures and potential health outcomes.

However, the results of the present study may have a number of potential limitations. It is possible the results observed may have occurred from unknown biases or cofounders present in the datasets examined. This seems unlikely because other control outcomes (i.e. outcomes that are not biologically plausibly linked to postnatal organic-Hg exposure from Thimerosal-containing vaccines) were examined, such as a diagnosis of congenital anomalies (ICD-9 code: 759.9) in the VSD database, using the same methodology employed for ASD. No similar patterns of significant associations were observed as that found for organic-Hg exposure from Thimerosal-containing hepatitis B vaccine administration and the subsequent risk of ASD diagnosis. For example, exposure to 12.5 μg organic-Hg from Thimerosal-containing hepatitis B vaccines administered in the first month of life in comparison to 0 μg organic-Hg from Thimerosal-containing hepatitis B vaccines administered in the first month of life among cases and controls revealed similar risk of exposure (odds ratio = 1.03, p > 0.50). Similarly, adverse event reports with the control outcome symptom of febrile convulsion (VAERS code: 10016284) were searched for in VAERS using the same methodology employed for ASD. The results of that analysis failed to reveal a similar pattern of a significant association observed for organic-Hg exposure from Thimerosal-containing DTaP vaccine in comparison to Thimerosal-free DTaP vaccine administration and the risk of reported ASD. There were 48 adverse event reports with febrile convulsions reported to VAERS following administration of Thimerosal-containing DTaP vaccines in comparison to 41 adverse event reports with febrile conclusions reported to VAERS following administration of Thimerosal-free DTaP vaccines (risk ratio = 1.06, p > 0.50).

It is also possible that the VAERS and VSD databases examined in the present study may have limitations. As described previously, VAERS may have shortcomings, such as underreporting, difficulty in determining causal relationship, lack of precise denominators. Nevertheless, as previously described by investigators from the CDC, almost all of these types of shortcomings would apply equally to VAERS reports after vaccines administered to similar populations [32], such as the Thimerosal-containing and Thimerosal-free DTaP vaccines examined in the present study. As a result, the comparison of vaccines administered to similar populations should provide accurate relative qualitative and quantitative relationships between vaccine exposures and adverse outcomes [32]. The VSD may also have limitations because despite having vaccination and medical records linked by computer, the cohort in VSD is restricted to a limited population derived from several different participating HMOs. The validity of the databases examined in the present study is significantly supported by the consistency of the results observed in VAERS and VSD.

Another potential limitation of the present study is that the results observed may be the result of statistical chance. However, such a possibility would be unlikely given the limited number of statistical tests performed, the highly significant results observed (most p-values observed were < 0.01), and the consistency in the direction and magnitude of the results observed in two separate epidemiological databases.

Still, other potential limitations of the present study include the possibilities that some of the individuals in the cohorts in VAERS and VSD examined may have had more subtle neurological dysfunction that was not brought to the attention of their healthcare providers, healthcare providers may have misdiagnosed some individuals, or some vaccine exposures may not have been appropriately classified. These limitations, while possibly present in the data examined in the current study, should not have significantly impacted the results observed because it is unclear how differential application would have occurred to the study cohorts examined based upon the Thimerosal doses that the individuals received. Moreover, misclassification occurring in the data examined would tend to bias any results observed toward the null hypothesis, since such effects would result in individuals being placed in the wrong exposure and/or outcome categories examined, and result in decreased statistical power to determine true potential exposure-outcome relationships.

In addition, another potential limitation of the present study is that exposures to other sources of Hg were not evaluated. It is very likely that the individuals examined in the present study incurred other organic-Hg exposures from other Thimerosal-containing childhood vaccines, dental amalgams, fish, or other environmental sources. While these other sources of Hg may play a significant involvement in the pathogenesis ASD, these unaccounted for Hg exposures would actually tend to bias the results observed towards the null hypothesis because they potentially would confound the specific exposure classifications of Hg examined. For example, individuals classified as having lower organic-Hg exposure from Thimerosal-containing vaccines may have actually received high doses of Hg from other sources, and individuals having higher organic-Hg exposure from Thimerosal-containing vaccines may have actually received low doses of Hg from other sources, with the net result tending to minimize the magnitude of the associations observed.

It is also possible that the findings may be the result of the vaccines themselves or other components of the vaccines studied, which in isolation or synergistically, interacted with the organic-Hg exposures examined in the present study. The former seems remote, since in our study of DTaP vaccines in VAERS for example, as summarized in Table 3, the composition of the Thimerosal-free DTaP vaccine in comparison to the Thimerosal-containing DTaP vaccine indicates that vaccine components such as antigens, aluminum, formaldehyde, and other trace constituents were more present overall in the Thimerosal-free DTaP vaccine in comparison to the Thimerosal-containing DTaP vaccine. We suggest that in future studies additional examination should be undertaken further evaluate the potential adverse consequences of the vaccines themselves or other vaccine components both as a function of exposure and timing of exposure.

**Table 3:** The composition of the DTaP vaccines under study in the VAERS

**Vaccine component**	**Thimerosal**-**containing DTaP vaccine (Pfizer\Wyeth-Lederle)**	**Thimerosal-free DTaP vaccine (GlaxoSmithKline)**
Pertussis toxin (μg/dose)	3.5	25
Filamentous hemagglutinin (μg/dose)	35	25
Pertactin (μg/dose)	2	8
Fembrial agglutinogens (μg/dose)	0.8	-
Diphtheria toxoid (Lf/dose)	9	25
Tetanus toxoid (Lf.dose)	5	10
How toxoided	Formaldehyde	Formaldehyde
Aluminum (mg/dose)	0.23	0.50
Diluent	Phosphate-Buffered Saline	Saline
Preservative	Thimerosal	Phenoxyethanol
Trace constituents	Formaldehyde, Gelatin, Polysorbate-80	Formaldehyde, Polysorbate-80

Another limitation of the present study is that individuals diagnosed with an ASD were based upon the information contained in the automated adverse event reports reported to VAERS or in the ICD-9 codes recorded within the automated medical records in VSD. As such, no additional medical information was provided about the individuals examined, such as whether the individuals diagnosed with an ASD experienced a regression. As discussed previously, it would be important to examine such phenomena in future studies, since regressive ASD would be expected to be associated with a neurological insult occurring in the early postnatal period, whereas non-regressive ASD would be expected to be more associated with a neurological insult occurring in the prenatal period. It would be worthwhile in future studies to attempt to further examine the potential relationship between timing of Hg exposure and the initial onset of ASD symptoms.

Finally, the current study suffers from the potential limitation that analyses were not conducted to further explore the precise timing and cumulative doses of organic-Hg from all Thimerosal-containing childhood vaccines associated with maximum adverse consequences. In future studies it would be worthwhile to further explore these precise-timing and cumulative-doses phenomena. In addition, it would be valuable to evaluate other neurodevelopmental outcomes, as well as other covariates such as gender, race, birth weight, etc., that may further affect the magnitude of the adverse effects found.

## Conclusion

The present study provides new epidemiological evidence supporting an association between increasing organic-Hg exposure from Thimerosal-containing childhood vaccines and the subsequent risk of ASD diagnosis. Using a two-phased, hypothesis-generating and hypothesis-testing, epidemiological analytical methodology in two separate databases, organic-Hg exposure from Thimerosal-containing childhood vaccines was determined to be associated with a subsequent diagnosis of an ASD. In addition, in phase II within the VSD, the present study placed special emphasis on requiring an adequate follow-up period in the analysis. Thus, the cases and controls were followed for a sufficient, evidenced-based interval of time, to ensure that they were appropriately classified with respect to their exposures and outcomes, thus helping to ensure the potential for a cause-and-effect relationship between exposure and outcome was not biased or confounded. Future studies should be completed to further evaluate the relationship between other sources of organic-Hg exposure from Thimerosal-containing childhood vaccines and other chronic disorders, and to further explore potential subpopulations and the timing of exposure to organic-Hg from Thimerosal-containing vaccine administration associated with adverse outcomes.

As previously described, routine childhood vaccination is an important public health tool to reduce the morbidity and mortality associated with infectious diseases. However, it is also a public health imperative to end the unnecessary addition of organic-Hg to vaccines in the form of Thimerosal used as a preservative, based on data showing an association between its administration and adverse outcomes.

## Abbreviations

(ACIP): Advisory Committee on Immunization Practices; (ASD): Autism spectrum disorder; (BSS): Biologics surveillance summaries; (DTaP): Diphtheria-Tetanus-acellular-Pertussis; (IRB): Institutional Review Board; (KPC): Kaiser Permanente Colorado; (KPNW): Kaiser Permanente North-West; (KPNC): Kaiser Permanente Northern California; (Hg): mercury; (NHIS): National Health Interview Survey; (NIP): National Immunization Program; (CDC): US Centers for Disease Control and Prevention; (FDA): US Food and Drug Administration; (VAERS): Vaccine adverse event reporting system; (VSD): Vaccine safety datalink.

## Competing interests

All of the investigators on the present study have been involved in vaccine/biologic litigation.

## Authors’ contributions

DG was the main writer and analyzed data, BH was the main computer programmer and reviewed the manuscript structure, ideas and science, JK reviewed the manuscript structure, ideas and science, PG reviewed manuscript structure, ideas, and science, LK edited manuscript structure, and MG evaluated and reviewed manuscript ideas and science. All authors read and approved the final manuscript.
